# Family psychological capital and adolescent academic burnout in Shanghai: the moderating role of spatial stratification

**DOI:** 10.3389/fpubh.2026.1814753

**Published:** 2026-08-19

**Authors:** Qingyu Bu, Mei Peng, Siyan Wen, Can Yang, Zhuojun Yu, Shiyi Gao, Yuncong Yang

**Affiliations:** 1 School of Philosophy and Law and Political Science, Shanghai Normal University, Shanghai, China; 2 School of Mental Health, Wenzhou Medical University, Wenzhou, China; 3 Faculty of Psychology, Southwest University, Chongqing, China; 4 School of Psychology and Cognitive Science, East China Normal University, Shanghai, China; 5 School of Public Administration, Guangdong University of Foreign Studies, Guangzhou, China

**Keywords:** academic burnout, intergenerational transmission, psychological capital, social capital, urban spatial stratification

## Abstract

In many emerging countries, rapid urbanization has intensified spatial inequality in metropolitan areas, which has consequently altered mechanisms of family interaction. To be specific, mechanisms such as family psychological capital have strong influence towards adolescents’ academic development. However, little is known about how both social spatial inequality and family psychological traits jointly affect students’ academic development in such urban contexts. Focusing on adolescents’ academic burnout, in our study we adopted moderated mediation models to clarify how spatial hierarchy influences the impact of family psychological factors on academic development in the megacity of Shanghai. Using a survey questionnaire, we collected data from 501 families with an adolescent student and their two parents (*n* = 1,503). The results indicate that family psychological capital can significantly reduce adolescents’ academic burnout and that spatial stratification can significantly moderate parent–child communication. Although community social capital (e.g., social networks, resources, and information access) did not significantly contribute to those relationships, the effect was significantly stronger in urban than suburban areas in Shanghai. In general, the spatial hierarchy based on urbanicity provides more advantages than social networks when using an intergenerational psychological pathway to reduce academic burnout among adolescents.

## 1 Introduction

In large cities, academic burnout among adolescents has become an increasingly prominent factor of their mental health and academic development (1, 2). Among the various factors influencing adolescents’ academic burnout, the family environment and role of the parents are especially critical (3). Empirical evidence indicates that the family serves as the core environment shaping adolescents’ academic development, with parents’ psychological states and interaction styles playing a decisive role (4, 5). On the one hand, positive parent–adolescent interactions, including conveying high expectations (5) and engaging effectively (4), can provide essential psychological support and form an important foundation for adolescents’ academic success and positive attitudes. On the other, negative psychological states among parents can exert equally profound detrimental effects (6). In such cases, parent–adolescent interaction styles can be improved through scientifically based intervention programs and concurrently enhance adolescents’ academic and social–emotional competencies (7).

The literature addressing how parent influences adolescents’ academic developments often concentrate on the impact of intergenerational factors according to the psychological model *parental characteristics → parent–adolescent interaction → adolescents’ outcomes* (8–10). However, when clarifying that model for academic burnout among adolescents, the literature just as often ignores the sociological impact of urban spatial inequality resulting from rapid urbanization. Such neglect is unjustified, for urban spatial inequality, originating from urban planning policies, has resulted in the unequal distribution of educational resources (11, 12). In turn, such inequality in educational resources could profoundly shape the mentioned model (8, 10). Despite that likelihood, the ways in which intergenerational psychological factors and urban spatial sociological factors jointly affect adolescents’ academic burnout remain unexamined.

Thus, in our study, integrating Bowen’s family systems theory (13) with Bronfenbrenner’s (14) ecological systems theory, we conceptualized family psychological capital as a transmissible resource. From that standpoint, we examined its influence on academic burnout among adolescents via parent–child communication by investigating the pathway *parental characteristics → parent–child interaction → adolescents’ academic burnout*. In addition, by simultaneously considering community social capital and spatial stratification as sociological moderators indicating urban spatial inequality, we sought to illuminate adolescents’ academic development under the influence of family psychological interaction and spatial inequality in a metropolitan context.

Using a quantitative questionnaire, we collected more than 1,500 responses via survey from participants living in Shanghai, China, a prototypical megacity within newly industrialized countries. Within a single generation, Shanghai’s market-driven development has intensified educational inequality alongside spatial disparities (15). Empirical evidence from Chinese cities indicates pronounced sociospatial stratification, with core districts that possess markedly stronger educational resources than peripheral areas (8). Such resource inequality directly influences family strategies and access to quality education (15). Moreover, residents in Shanghai’s outlying districts often experience longer, more stressful commutes, which substantially diminish psychological well-being (16). Recent research on families in Shanghai has also shown that maternal distress and parenting stress are associated with maladaptive parenting and children’s problematic media use (17). Thus, Shanghai functions as an important site for studying multilevel influences on adolescents’ development in the context of rapid urbanization.

## 2 Literature

### 2.1 Intergenerational psychological perspective

Drawing from Bowen’s family systems theory (13), this section examines how family psychological capital influences adolescents’ academic burnout through the family projection process. The theory posits that parental psychological resources are transmitted as integrated emotional–cognitive–behavioral patterns that shape their children’s coping models for academic stress.

#### 2.1.1 Family psychological capital and adolescents’ academic burnout

In Bowen’s family projection process, family psychological capital, encompassing hope, self-efficacy, optimism, and resilience (18), serves as the input resource. According to family systems theory, capital is not transmitted as an isolated trait but is an integrated emotional–cognitive–behavioral pattern that children internalize through observational learning (13). Moreover, that process directly shapes their capacity to cope with academic stress and their susceptibility to burnout.

When family psychological capital is high, parents demonstrate differentiated functioning under stress. They regulate emotions before discussing strategies, turn failure into learning opportunities, and persistently co-set goals with children. Those behaviors constitute a supportive communication style—calm, constructive, and collaborative—that children replicate as part of adaptive coping models. That pathway has been empirically validated; parental warmth, a marker of high capital, has been shown to enhance children’s emotional stability (19) and academic self-efficacy (20) and, in turn, to directly reduce burnout.

By contrast, low psychological capital, manifesting as reactive, anxious communication, erodes differentiation. Parents transmit stress through emotional volatility, through catastrophic attributions (interpreting a setback such as a poor test score as a sign of permanent, across-the-board failure), and through fragmented problem-solving (reacting to academic difficulties in piecemeal, inconsistent ways rather than with a coherent strategy). This compromises parent–child communication and leads children to internalize maladaptive coping patterns. Research has confirmed the chain reaction in which parental stress diminishes children’s resilience and heightens their anxiety (21), parental burnout escalates risk through harsh parenting (22), and educational anxiety disrupts family functioning (6). Thus, family psychological capital may shape the quality of parent–child communication.

#### 2.1.2 Mediating role of parent–child communication

In Bowen’s theory, the primary vehicle for the family projection process is parent–child communication (23), which involves bidirectional emotional exchange and family members’ co-construction of meaning (24). When family psychological capital is high, parents engage in differentiated communication—calm, constructive, and supportive—that transmits adaptive coping models to children. Conversely, low capital, manifesting as psychological depletion, undermines communication and indirectly elevates the risk of burnout (56). Likewise, parental conflict damages the openness of relationships, thereby eroding effective communication and exacerbating adolescent burnout (25). Those findings are consistent with the proposition that the quality of communication mediates the Bowenian transmission pathway.

High-quality communication functions as a mechanism for negotiating meaning that buffers burnout cognitively, emotionally, and behaviorally. Cognitively, parents provide scaffolding for positive thoughts about learning (26, 27); emotionally, empathetic responses foster secure attachment and emotion regulation (28); and behaviorally, open dialogue reframes stress and co-develops coping strategies (29). By contrast, parental depletion or conflict, in disrupting those functions, corrodes the openness and support necessary for adaptive internalization.

Within Bowen’s framework, psychological capital’s dimensions enhance communication differentially. Resilience maintains emotional stability under stress, which reduces conflict (30); self-efficacy promotes constructive strategies that correlate strongly with the quality of communication (31); and hope motivates encouraging messages that enhance openness (32). Theoretically, *family psychological capital → parent–child communication → adolescent burnout* may represent a key mediation pathway through which intergenerational transmission operates. However, empirical verification is required to establish whether parent–child communication functions as the core mechanism linking capital to burnout-related outcomes.

### 2.2 Community sociological perspective

Although Bowen’s family systems theory illuminates intra-family transmission mechanisms, it overlooks how educational inequality at the community level shapes those processes. Bronfenbrenner’s (14) ecological systems theory narrows that gap by conceptualizing the community as a resource transfer station that unequally distributes educational opportunities. In China’s quasi-market education system, community social capital and spatial stratification constitute structural sources of educational inequality. In determining access to high-quality schools, extracurricular resources, and peer networks, they create unequal starting points for adolescents regardless of family-level psychological capital (10, 15). The following sections examine how those two dimensions of educational inequality moderate the efficacy of converting family psychological capital to parent–child communication.

#### 2.2.1 Moderating role of community social capital

*Community social capital*, defined as structural resources embedded in neighborhood networks (33), may moderate the strength of the relationship between family psychological capital and the quality of parent–child communication. Putnam’s (33) classic research has conceptualized that construct in three core dimensions: social networks (i.e., density of organizational or informal ties), social trust (i.e., mutual assistance predictability), and reciprocity norms (i.e., resource-sharing agreements). Together, those elements can form a community support system that potentially influences the conversion of parental capital into supportive interactions.

Empirical evidence suggests distinct mechanisms for moderation in each dimension. First, the social network’s structure may set the boundaries for capital conversion. Gu and Xiao (34) found that weak-tie networks can amplify psychological capital’s positive effects, whereas low-density networks can impede the translation of capital into communication skills. Second, low social trust is often accompanied by a heightened perception of risk, and Goldner et al. (35) have observed that in higher-risk communities, the association between parental monitoring and children’s externalizing behaviors was significantly stronger. That observation implies that low-trust environments may suppress or even distort the positive expression of parental optimism and self-efficacy, which weakens their promotive effect on communication. Third, reciprocity norms can function by releasing cognitive–emotional resources. For example, strong community involvement can reduce the costs of resource acquisition and free up parents’ capacity for child-rearing (36).

Collectively, those findings indicate that communities with abundant social capital may reduce educational inequality by enhancing the transmission of support from schools and public services to parents, thereby sustaining psychological capital for differentiated communication. Conversely, resource-poor communities may exacerbate educational disadvantages by depleting parental resilience and making communication reactive and less supportive. However, whether social capital moderates the specific pathway from family psychological capital to parent–child communication within educationally stratified contexts has seldom been empirically tested.

#### 2.2.2 Moderating role of spatial stratification

Spatial stratification, operationalized as the gradient of educational resource density across geographic locations (e.g., urban core vs. suburbs), may moderate the strength of the relationship between family psychological capital and parent–child communication. Residential differentiation exhibits a distinct “sociospatial coupling” (10); urban cores provide denser, highly accessible resources (e.g., quality schools and public services), whereas suburban and peripheral areas face scarcity and longer acquisition times (10). The disparity could shape the efficacy of converting parental capital into supportive communication via three mechanisms.

First, educational accessibility may vary spatially and thereby create unequal competitive advantages. Urban cores host high-performing schools and tutoring centers in high concentration, which requires less effort from parents in procuring them as resources. Suburban parents might invest more time and finances in accessing those services, which may deplete the psychological capital available for quality communication (10). Second, time costs, manifested as lengthy commutes, may erode time for family-adolescents’ interaction and increase emotional exhaustion. Third, access to educational information may differ. The gap lies not in internet connectivity, which is broadly available, but in the localized, often tacit knowledge that circulates through interpersonal networks. Much of what helps families navigate a competitive system travel by word of mouth among parents, teachers, and neighbors: which schools and tutors are effective, how admissions actually work, and when to act. Urban cores offer denser, more education-rich networks that expose parents to such knowledge sooner, whereas more peripheral suburban parents may encounter beneficial resources later or not at all.

Those constraints intensify emotional pressures. Suburban parents experiencing “time poverty,” so to speak, might exhibit elevated emotional thresholds, which increases the family’s susceptibility to conflict (25). According to Qin et al. (37), spatial separation reduces the quality of emotional communication and may thus suppress the protective effects of parental resilience and optimism. Conversely, high-resource urban cores may create structural advantages; abundant services may reduce parenting costs, thereby freeing up parents’ capacity for attentive communication (38).

In sum, spatial stratification theoretically moderates the pathway from parental capital to communication by amplifying or mitigating educational inequality. High-resource urban cores may compensate for deficiencies in family psychological capital, whereas low-resource suburbs may intensify its depletion and thus reproduce educational disadvantage across generations. However, that specific moderation effect needs rigorous empirical testing.

## 3 Research questions

### 3.1 Research gap

Research on parent–child relationships and adolescents’ development has emphasized negative psychological traits and stressors across generations while focusing less on the intergenerational transmission of positive psychological resources. Although correlations between parental well-being and children’s outcomes have been established, the specific mechanisms of family psychological capital (e.g., hope, efficacy, resilience, and optimism) influencing adolescent academic functioning remain insufficiently explored. Moreover, the literature exhibits a significant gap in knowledge about how that potential transmission pathway is simultaneously shaped by different layers of the ecological environment. In particular, the joint moderating roles of community social capital, representing relational resources, and spatial stratification, representing structural inequalities, remain largely unexamined. In our study, we address that critical gap by integrating Putnam’s (33) social capital theory with Bronfenbrenner’s (14) ecological systems theory to construct a comprehensive framework. The framework allows a systematic investigation into how community environments and spatial structures condition the process through which family psychological capital is translated into effective parent–child communication and, in turn, influence academic burnout among adolescents. Those findings can offer empirical evidence for context-sensitive community interventions that enhance family interaction.

### 3.2 Research questions and hypotheses

Guided by an intergenerational perspective on positive psychological resources, we aimed to achieve three primary objectives. First, we examined the direct relationship between family psychological capital and adolescent academic burnout. Second, we investigated the mediating role of parent–child communication in that relationship, and third, we explored the moderating effects of community social capital and spatial stratification on the pathway from family psychological capital to parent–child communication. For theory, our research contributes to the literature by extending intergenerational transmission theory into the domain of positive family dynamics and by contextualizing family systems theory within the specific sociospatial realities of urban China. For practice, the findings provide a scientific basis for developing targeted family support programs and evidence-based recommendations for community-level initiatives. To achieve those objectives, we formulated three research questions (RQs) and accompanying hypotheses (H).

*RQ1:* What is the relationship between family psychological capital and adolescents’academic burnout?

*H1:* Family psychological capital is negatively associated with adolescents’ academic burnout.

RQ1 inquires into the direct relationship between parental psychological resources and adolescents’ educational outcomes. Within the family system, parental forms of psychological capital (e.g., hope, efficacy, resilience, and optimism) directly shape adolescents’ academic experiences. Parents with higher psychological capital are better equipped to model adaptive coping strategies and create a home environment that buffers against academic burnout. That theoretical perspective is grounded in the conservation of resources theory (21, 39), which emphasizes how resource dynamics influence family functioning.

*RQ2:* How does parent–child communication influence the relationship between family psychological capital and adolescents’ academic burnout?

*H2:* Parent–child communication mediates the relationship between family psychological capital and adolescents’ academic burnout.

RQ2 investigates the mediating mechanism underlying the relationship articulated in RQ1. Drawing on family systems theory (23), we conceptualized parent–child communication as the primary channel through which psychological capital is transmitted (26). Such communication, as a means for negotiating meaning, provides cognitive scaffolding and emotional support (30, 31). Parents with higher psychological capital are more likely to engage in constructive communication patterns that mitigate academic burnout (25).

*RQ3:* How do community social capital and spatial stratification influence the relationship between family psychological capital and parent–child communication?

*H3a:* Community social capital positively moderates the strength of the effect of family psychological capital on parent–child communication.

*H3b:* Spatial stratification significantly moderates the effect of family psychological capital on parent–child communication.

Last, RQ3 explores the contextual boundaries of the mechanism in RQ2 via ecological moderators (14). Community contexts function as resource transfer stations where social capital is exchanged through networks (34), trust (35), and reciprocity (36). Simultaneously, spatial stratification, in creating unequal access to resources and shaping patterns of interaction, positions geographical location as a key dimension that influences family processes (10).

## 4 Data and measurements

### 4.1 Data collection

For our study, we administered a survey questionnaire and collected responses from 604 families with adolescents 15–18 years old attending eight middle schools and high schools. All eight schools were controlled for their academic ratings and evenly distributed across three road “ring” loops in Shanghai. The locations of three road ring loops are indicated in their names (i.e., “inner,” “middle” and “outer”). In each family, three family members completed the questionnaire: the student, the mother, and the father. After data cleaning, 501 families, with 1,503 valid questionnaires in all, were retained for analysis. All participating parents hold at least a bachelor’s degree, and each parental couple lived in the same household regardless of marital status. Because family psychological capital was operationalized as the combined psychological resources of both a mother and a father (see Section 4.3), eligibility was restricted at recruitment to two-parent households in which both parents resided with the adolescent. Single-parent and other family forms were not recruited, as the dyadic parental measure at the core of our framework cannot be constructed when one parent is absent. This criterion follows Bowen’s treatment of the family as an interdependent emotional unit (13), in which the joint functioning of the parental subsystem—rather than either parent alone—shapes the adolescent’s coping environment.

To address the RQs, the questionnaire included variables related to the original psychological pathway (i.e., family psychological capital, parent–child communication, and adolescents’ academic burnout), sociospatial moderators, and the household’s spatial stratification (i.e., urban vs. suburban). Table 1 shows the information of the participants. Table 2 presents the descriptive statistics for all model variables before standardization.

**Table 1:** Information of participants.

Information of participating families	Categories	Number of families
Teenagers’ gender	Male	258
	Female	243
Teenagers’ grader	Grade 10	170
	Grade 11	160
	Grade 12	171
Spatial stratification	Urban	248
	Suburban	253

**Table 2:** Descriptive statistics of study variables.

Variable	M	SD	Min	Max
Father psychological capital	133.30	14.63	64	167
Mother psychological capital	129.97	14.18	57	166
Family psychological capital	263.26	22.69	142	316
Father–child communication	69.82	13.96	20	90
Mother–child communication	72.83	11.14	20	90
Parent–child communication	142.65	23.53	49	180
Adolescent academic burnout	42.59	5.96	29	61
Community social capital	200.80	64.80	45	460

### 4.2 Dependent variables

Academic burnout was measured using Wu et al. (40, 41) Adolescent Academic Burnout Scale, a 16-item instrument with three subscales—Academic Alienation, Physical and Mental Exhaustion, and Reduced Self-Efficacy—that correspond to its three theoretical dimensions. Participants indicated their agreement with the items on a 5-point Likert scale, with higher composite scores indicating more severe manifestations of academic burnout. The scale has demonstrated satisfactory psychometric properties in previous studies in China involving adolescents and maintained good reliability in our study. In the present sample, Cronbach’s *α* was 0.83, indicating good reliability.

### 4.3 Independent variables

To capture collective resources in the parental subsystem, we constructed a composite measure called “family psychological capital” that represented the sum of the psychological capital scores of both fathers and mothers. The resulting family-level composite was subsequently standardized before model estimation. That approach conceptualizes the family as an interconnected emotional unit wherein the psychological resources of individual members mutually influence one another to shape the family’s overall psychological environment (42).

The psychological capital of individual fathers and mothers was assessed using the 26-item Positive Psychological Capital Questionnaire (43), which has established reliability and validity in Chinese populations. It measures four dimensions—self-efficacy, hope, resilience, and optimism—on a 7-point Likert scale ranging from 1 (*strongly disagree*) to 7 (*strongly agree*); thus, a higher score indicated a greater abundance of positive psychological resources within the family system. In the present sample, Cronbach’s α was 0.86 for fathers and 0.87 for mothers, indicating good internal consistency.

Because a simple sum of paternal and maternal scores may be sensitive to systematic differences in their score distributions, we additionally constructed an alternative family psychological capital composite for sensitivity analysis. Specifically, paternal and maternal total psychological capital scores were standardized separately and then averaged:


$$\text{Family psychological capital}=\frac{\begin{array}{l} Z(\text{Fathers}\;\text{Psychological Capital})\\ +Z(\text{Mothers}\;\text{Psychological Capital}) \end{array}}{2}$$

This procedure assigned equal weight to each parent on a parent-specific standardized metric. The original summed composite remained the focal measure in the main analyses, whereas the alternative composite was used only to evaluate the robustness of the findings.

### 4.4 Mediating variable

To elucidate how family psychological capital translates into reduced academic burnout in adolescents, we employed a mediating variable, parent–child communication, from family systems theory, which posits that internal psychological resources externalized through processes of family interaction shape adolescents’ development.

The quality of parent–child communication was assessed with the Parent-Adolescent Communication Scale (PACS), originally developed by Barnes and Olson (44) and adapted for Chinese adolescents by An (45). The instrument captures communication along two dimensions, each measured by a 10-item subscale. The Open Communication subscale assesses how freely and comfortably family members exchange information and express emotions (e.g., “I find it easy to discuss problems with my parents”), whereas the Problematic Communication subscale assesses barriers, hesitancy, and topic avoidance in family exchanges (e.g., “I avoid discussing certain topics with my parents”). All 20 items used a 5-point Likert format ranging from 1 (strongly disagree) to 5 (strongly agree). The 10 Problematic Communication items were reverse-coded so that higher composite scores indicate more open and higher-quality communication. The Chinese adaptation has demonstrated sound reliability and validity in adolescent samples (45).

Adolescents completed the Parent–Adolescent Communication Scale twice, once with reference to communication with their father and once with reference to communication with their mother. This procedure yielded two relationship-specific assessments within each family. Composite scores for communication with the father and communication with the mother were calculated separately and then summed to create the family-level parent–child communication score used in the analyses. In the present sample, both versions demonstrated good internal consistency, with Cronbach’s *α* = 0.86 for communication with the father and α = 0.83 for communication with the mother.

### 4.5 Moderating variables

A moderating variable determines the conditions under which an independent variable’s effect on a dependent variable is strengthened or weakened. According to Bronfenbrenner’s (14) ecological systems theory, adolescents’ development is shaped by the interaction between individuals and multiple environmental systems, ranging from immediate microsystems to broader macrosystems (46). Thus, to identify contextual boundaries that may alter the strength of the relationship between family psychological capital and adolescent outcomes, we incorporated two moderating variables: spatial stratification and community social capital.

### 4.6 Measurement of spatial stratification

Consistent with established paradigms for analyzing sociospatial structures in Chinese cities (10, 15), we operationalized the variable of spatial stratification using a dichotomous classification (i.e., urban vs. suburban) based on the administrative and functional demarcation of the residential area. In general, the approach captures fundamental disparities in resource access, developmental priority, and socioeconomic composition between core urban zones and peripheral suburban districts. In our study, households within the primary urban districts—characterized by denser infrastructure, superior public service allocation, and higher housing market valuations—were coded “1” (i.e., urban group). Conversely, households in officially designated suburban counties or outer-ring territories, which typically exhibit less concentrated public resources and different community profiles, were coded “0” (i.e., suburban group). That binary distinction reflects a key dimension of spatial inequality, which allowed examining how a family’s position within the urban–suburban hierarchy moderates the psychosocial processes under investigation.

### 4.7 Measurement of community social capital

Community social capital was assessed using the Chinese Community Social Capital Measurement Indicators developed by Gui and Huang (47). The instrument comprises 29 mixed-format indicators across seven domains: local networks, community sentiment, community cohesion, non-local sociability, volunteerism, reciprocity and general trust, and community trust. Item formats include open-ended numerical responses, binary responses, and 4- and 5-point ordinal ratings. Following the original scoring procedure, the coded indicators were combined into a composite score, with higher scores indicating greater household community social capital. Because the open-ended numerical items differed substantially in response format and measurement scale, only the closed-ended items were included in the internal consistency analysis; Cronbach’s *α* was 0.82.

Unlike psychological capital, which reflects each parent’s individual internal states and was therefore assessed separately for fathers and mothers, community social capital captures the household’s shared embeddedness in neighborhood networks, trust, and reciprocity. In the Chinese cultural context, community participation and social ties tend to be organized at the household rather than individual level (47, 48). Each family accordingly designated either the father or the mother as the household informant to complete the community social capital questionnaire, a procedure common in household-based social surveys. All dependent, independent, moderating, and mediating variables appear in Table 3.

**Table 3:** List of variables.

No.	Dependent variables	No.	Independent variables
1	Adolescent academic burnout	1	Family psychological capital
	Mediation variable		Moderator variable
1	Parent–Child communication	1	Spatial stratification
		2	Community social capital

## 5 Methods

### 5.1 Analytical approach

In this study, we employed a moderated mediation framework to examine whether the indirect association between family psychological capital and adolescent academic burnout through parent–child communication varied across different contextual conditions. This framework was appropriate because our hypotheses involved both a mediating mechanism and contextual moderators. Specifically, parent–child communication was specified as the mediator, whereas spatial stratification and community social capital were specified as moderators of the first-stage path from family psychological capital to parent–child communication.

Data analysis was conducted using SPSS 27.0 and the PROCESS macro developed by Hayes (49). After preliminary checks for common method bias using Harman’s single-factor test, Pearson correlation analyses were conducted to examine bivariate associations among the study variables. PROCESS Model 4 was used to test the mediation model, and PROCESS Model 7 was used to test the first-stage moderated mediation models. Bias-corrected bootstrap confidence intervals were estimated using 5,000 resamples. To reduce multicollinearity and facilitate interpretation of interaction terms, all continuous variables were standardized before analysis.

**Figure 1:**
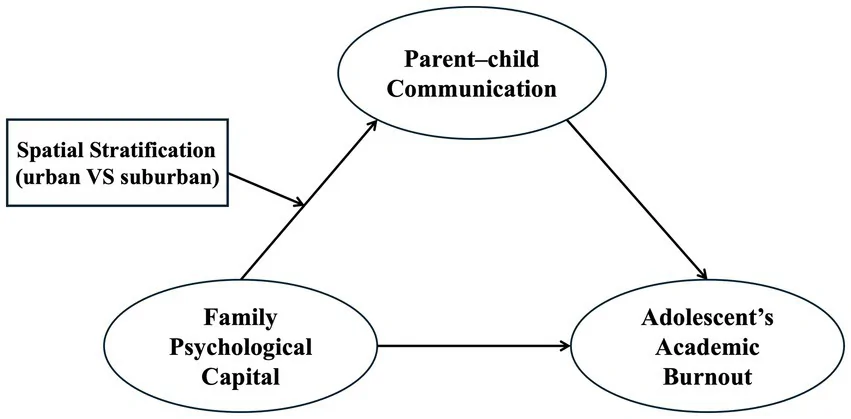
Conceptual model of the moderated mediation effect of spatial stratification.

**Figure 2:**
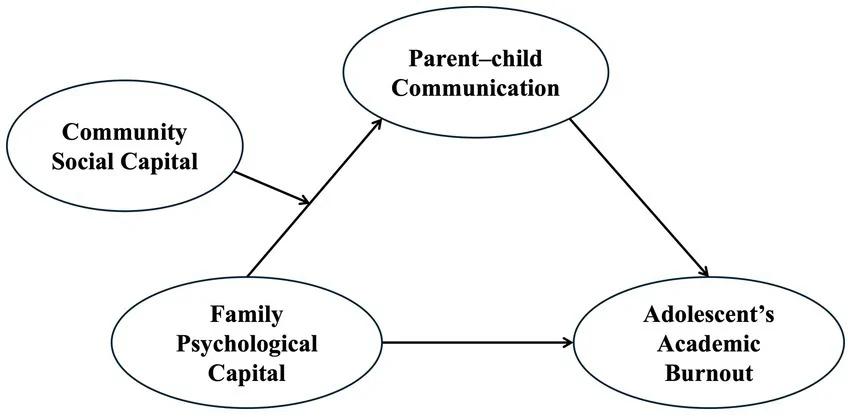
Conceptual model of the moderated mediation effect of community social capital.

Two moderated mediation models were estimated separately. Model 1 tested whether spatial stratification, coded as urban versus suburban residence, moderated the association between family psychological capital and parent–child communication. Model 2 tested whether community social capital moderated the same first-stage path. Estimating these models allowed us to examine whether the family-level psychological pathway linking family psychological capital to adolescent academic burnout operated differently across spatial and community contexts (see Figures 1, 2).

To examine whether the two contextual moderators retained their respective effects when estimated simultaneously, we also specified a unified moderated mediation model using PROCESS Model 9. Spatial stratification, community social capital, and their respective interaction terms with family psychological capital were entered into the mediator equation simultaneously.

To address the possibility that the family psychological capital composite was affected by systematic paternal–maternal score differences, paired-samples t tests were conducted to compare fathers and mothers on overall psychological capital and its four dimensions: self-efficacy, resilience, hope, and optimism. Holm-adjusted *p* values and paired-samples effect sizes (*d_z_*) were calculated. We then re-estimated the mediation model, the two separate moderated mediation models, and the unified moderated mediation model using the alternative composite based on the average of separately standardized paternal and maternal scores. The complete sensitivity analyses are reported in the Supplementary Material.

### 5.2 Models

The foundational mediation model examined the indirect pathway through which family psychological capital influences adolescents’ academic burnout via parent–child communication. The model was specified as follows:


$$\begin{array}{c} \text{Parent}–\text{child communication}=\alpha ₁+\beta ₁(\text{Family psychological capital})\\ +\varepsilon ₁ \end{array}$$

$$\begin{array}{c} {\text{Adolescents}}^{’}\;\text{academic burnout}=\alpha ₂+\beta ₂(\text{Family psychological capital})\\ +\beta ₃(\text{Parent}–\text{child communication})+\varepsilon ₂ \end{array}$$

The model, in establishing the basic relationships between the core constructs, tests the hypothesis that family psychological capital is associated with adolescents’ academic burnout by facilitating more effective parent–child communication.

To investigate the contextual boundaries of the established mediation pathway, we subsequently estimated two separate moderated mediation models, as follows:

#### 5.2.1 Model 1: spatial stratification as moderator

$$\begin{array}{c} \text{Parent}–\text{child communication}=\alpha ₁+\beta ₁(\text{Family psychological capital})\\ +\beta ₂(\text{Spatial stratification})\\ +\beta ₃(\begin{array}{l} \text{Family psychological capital}\\ \times \text{Spatial stratification} \end{array})\\ +\varepsilon ₁ \end{array}$$

$$\begin{array}{c} {\text{Adolescents}}^{’}\;\text{academic burnout}=\alpha ₂+\beta ₄(\text{Family psychological capital})\\ +\beta_{5}(\text{Parent}–\text{child communication})\\ +\varepsilon ₂ \end{array}$$

#### 5.2.2 Model 2: community social capital as moderator

$$\begin{array}{c} \text{Parent}–\text{child communication}=\alpha ₁+\beta ₁(\text{Family psychological capital})\\ +\beta ₂(\text{Community social capital})\\ +\beta ₃(\begin{array}{l} \text{Family psychological capital}\\ \times \text{Community social capital} \end{array})+\varepsilon ₁ \end{array}$$

$$\begin{array}{c} {\text{Adolescents}}^{’}\;\text{academic burnout}=\alpha ₂+\beta ₄(\text{Family psychological capital})\\ +\beta_{5}(\text{Parent}–\text{child communication})\\ +\varepsilon ₂ \end{array}$$

In both models, the interaction term, Family psychological capital × Moderator, tests whether the strength of the relationship between family psychological capital and the mediator, parent–child communication, systematically differs across different levels of the contextual moderator. To test the statistical significance of both the indirect and conditional indirect effects, we employed bias-corrected bootstrap confidence intervals (CI) with 5,000 resamples, which provided a robust inference for our hypothesized mechanisms.

## 6 Results

### 6.1 Family psychological capital and adolescents’ academic burnout (RQ1)

As presented in Table 4, the correlation analysis provided clear support for H1. The subsequent analysis of the correlations revealed a significant negative correlation between family psychological capital and adolescents’ academic burnout (*r* = −0.41, *p <* 0.001). That statistically robust association indicates that higher levels of family psychological capital corresponded to substantially lower levels of academic burnout among adolescents, which confirmed our initial proposition and established a foundational relationship for subsequent mediation and moderation analyses. The finding is also consistent with published results showing that psychological resources can buffer the outcomes related to stress (50). Altogether, those results, in confirming a significant negative association between family psychological capital and adolescents’ academic burnout, established the fundamental premise for subsequent analyses.

**Table 4:** Correlations between variables.

Variable	1	2	3	4	5
1. Family psychological capital	1				
2. Parent–child communication	0.717^***^	1			
3. Spatial stratification	−0.578^***^	−0.627^***^	1		
4. Community social capital	0.387^***^	0.235^***^	−0.212^***^	1	
5. Adolescent academic burnout	−0.411^***^	−0.378^***^	0.258^***^	−0.196^***^	1

*N* = 501. ^*^p < 0.05, ^**^p < 0.01, ^***^p < 0.001, the same applies below. Spatial stratification was a binary variable; its correlations with continuous variables are equivalent to point-biserial correlations.

### 6.2 Multicollinearity diagnostics

Before testing the hypothesized mediation and moderation effects, multicollinearity diagnostics were conducted for the mediator equations in both moderated mediation models and for their common outcome equation.

As shown in Table 5, in the spatial stratification model, tolerance values ranged from 0.207 to 0.633, VIF values ranged from 1.579 to 4.821, and the maximum condition index was 5.246. In the community social capital model, tolerance values ranged from 0.733 to 0.856, VIF values ranged from 1.168 to 1.364, and the maximum condition index was 1.836. In the common outcome equation, tolerance values were 0.486 and VIF values were 2.058, with a maximum condition index of 2.463. All tolerance values exceeded 0.20, all VIF values were below 5, and all maximum condition indices were below 10, indicating that substantial multicollinearity was not present in the estimated models.

**Table 5:** Multicollinearity diagnostics for the moderated mediation models.

Regression equation	Predictor	Tolerance	VIF	Maximum condition index
Spatial stratification model: mediator equation	Family psychological capital	0.207	4.821	5.246
	Spatial stratification	0.633	1.579	-
	Family psychological capital × spatial stratification	0.262	3.811	-
Community social capital model: mediator equation	Family psychological capital	0.733	1.364	1.836
	Community social capital	0.846	1.182	-
	Family psychological capital × community social capital	0.856	1.168	-
Common outcome equation	Family psychological capital	0.486	2.058	2.463
	Parent–child communication	0.486	2.058	-

### 6.3 Mediating role of parent–child communication (RQ2)

**Figure 3:**
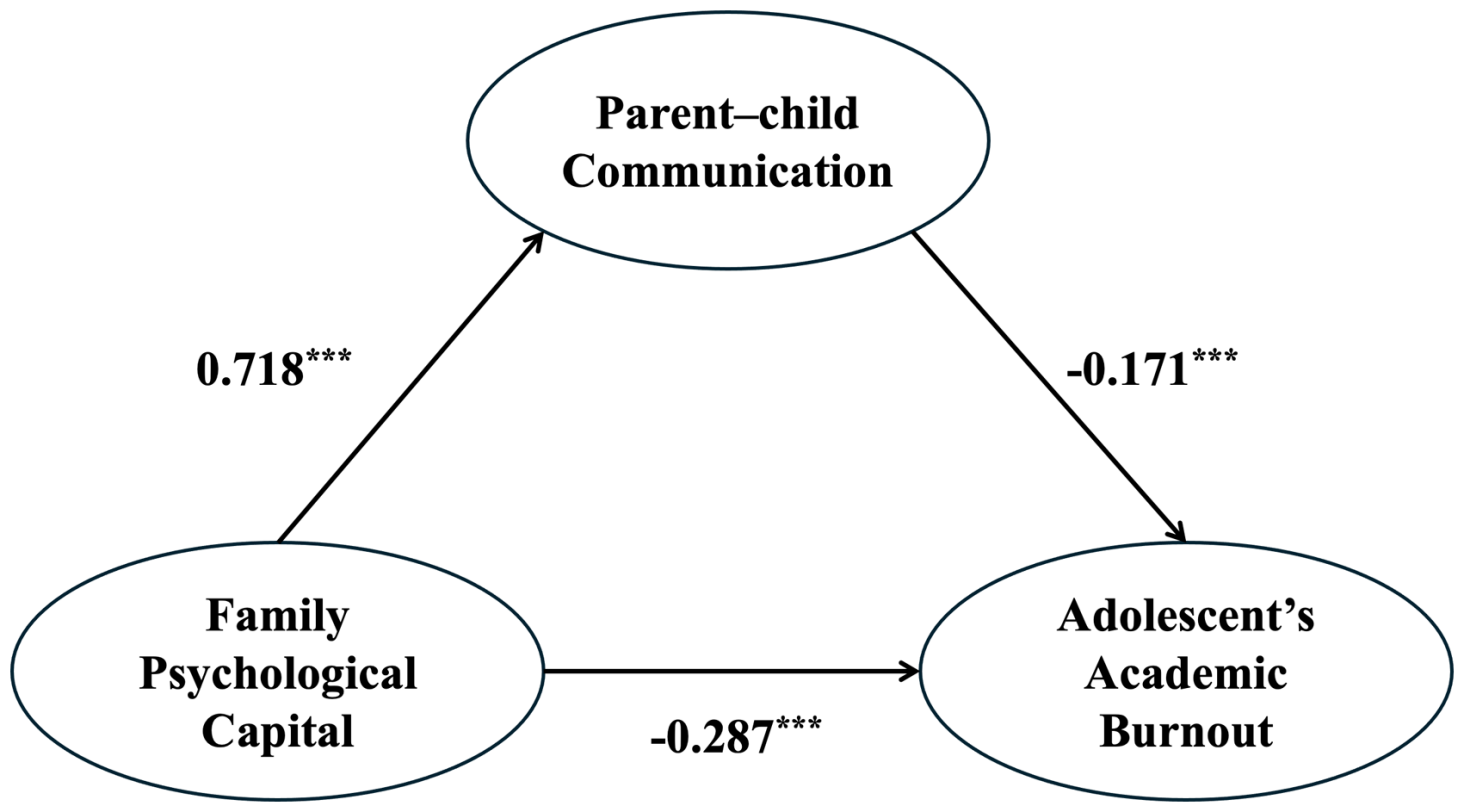
Mediating effect of parent–child communication between family psychological capital and adolescent academic burnout.

The mediation analysis (Table 6; Figure 3) provided strong support for H2. As summarized in Figure 3, the mediation analysis provided strong support for H2. The results demonstrate that family psychological capital significantly negatively predicted adolescents’ academic burnout (*B* = −0.287, *p <* 0.001) while showing a significant positive prediction for parent–child communication (*B* = 0.718, *p <* 0.001). When parent–child communication was subsequently included in the model, it emerged as a significant negative predictor of adolescents’ academic burnout (*B* = −0.171, *p =* 0.003).

**Table 6:** Mediating model test.

Model effect	Effect	SE	LLCI	ULCI
Total effect	−0.410	0.041	−0.490	−0.330
Direct effect	−0.287	0.058	−0.401	−0.173
Indirect effect	−0.123	0.049	−0.216	−0.026

The bootstrapping procedure further confirmed the mediating mechanism. The 95% CI for the total effect of family psychological capital on adolescents’ academic burnout was [−0.490, −0.330], while the 95% CI of the indirect effect through parent–child communication was [−0.216, −0.026] and the direct effect’s was [−0.401, −0.173]. None of those intervals included zero, which indicates statistically significant effects. Those findings collectively demonstrate that parent–child communication partly mediates the relationship between family psychological capital and adolescents’ academic burnout, which confirmed H2. Those findings align with family systems perspectives (51) by demonstrating how internal resources are externalized through family interactions to influence adolescents’ development. They also support a partial mediation model indicating that parent–child communication is one way in which family psychological capital mitigates adolescents’ academic burnout.

### 6.4 Moderating effect of community social capital (RQ3a)

**Figure 4:**
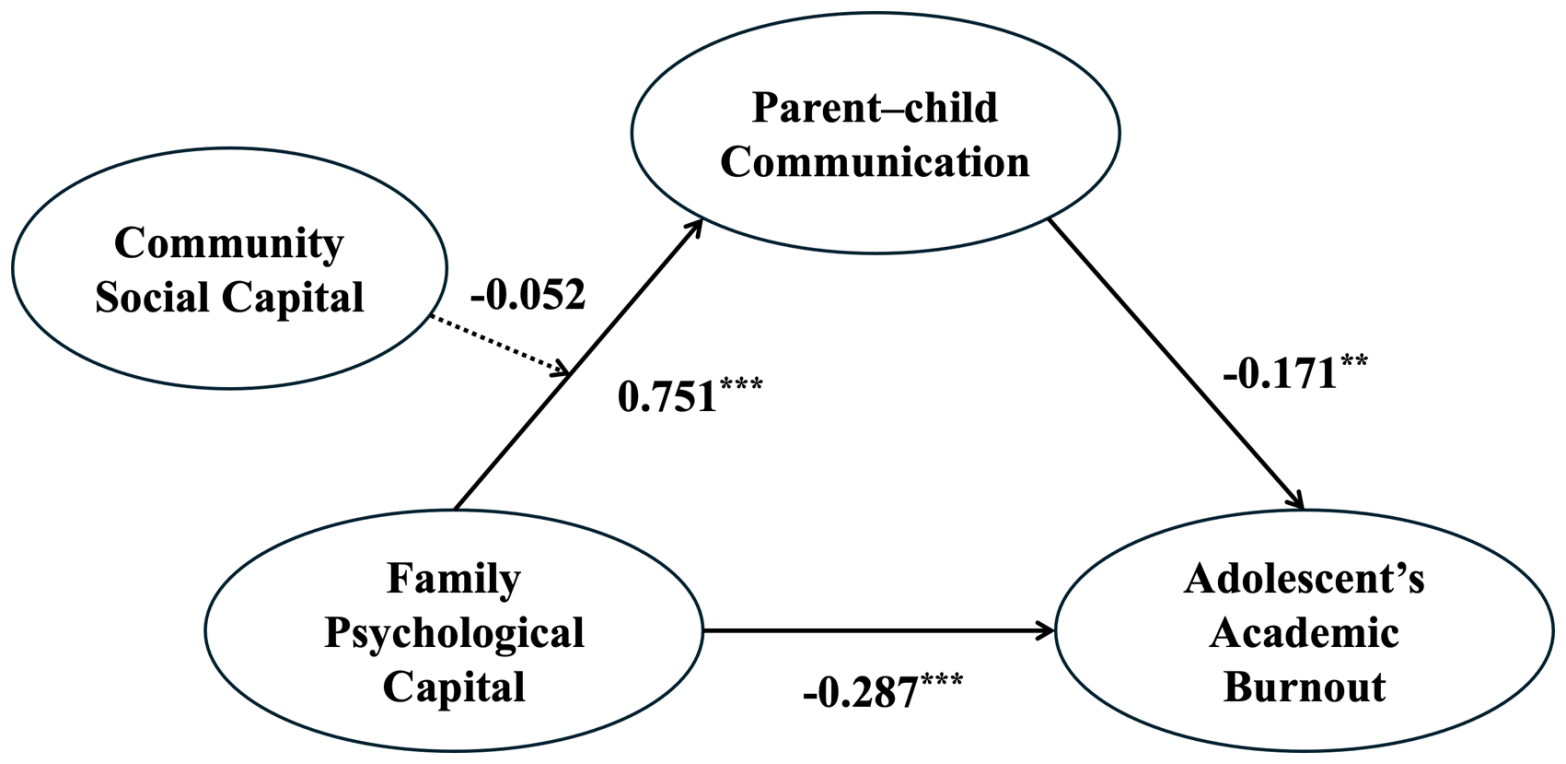
Moderated mediation model with community social capital as the moderator.

The moderated mediation analysis (Table 7; Figure 4) showed that family psychological capital was positively associated with parent–child communication (*B* = 0.751, *p* < 0.001). However, the interaction term between family psychological capital and community social capital did not reach statistical significance in predicting parent–child communication (*B* = 0.023, *p* = 0.329). This finding indicates that community social capital did not significantly moderate the association between family psychological capital and parent–child communication. Consequently, H3a, which postulated a positive moderating effect of community social capital on this specific pathway, was not supported. Overall, the results do not support the hypothesized moderating role of community social capital in the relationship between family psychological capital and parent–child communication.

**Table 7:** Moderated mediation model (community social capital).

Predictor variable	B	SE	t
Family psychological capital	0.751	0.036	20.59^***^
Community social capital	−0.052	0.034	−1.54
Family psychological capital * community social capital	0.023	0.024	0.98
Fitting indicators	*R*	0.72	
	*R^2^*	0.52	
	*F*	177.41^***^	

*p < 0.05, **p < 0.01, ***p < 0.001.

### 6.5 Moderating effect of spatial stratification (RQ3b)

**Figure 5:**
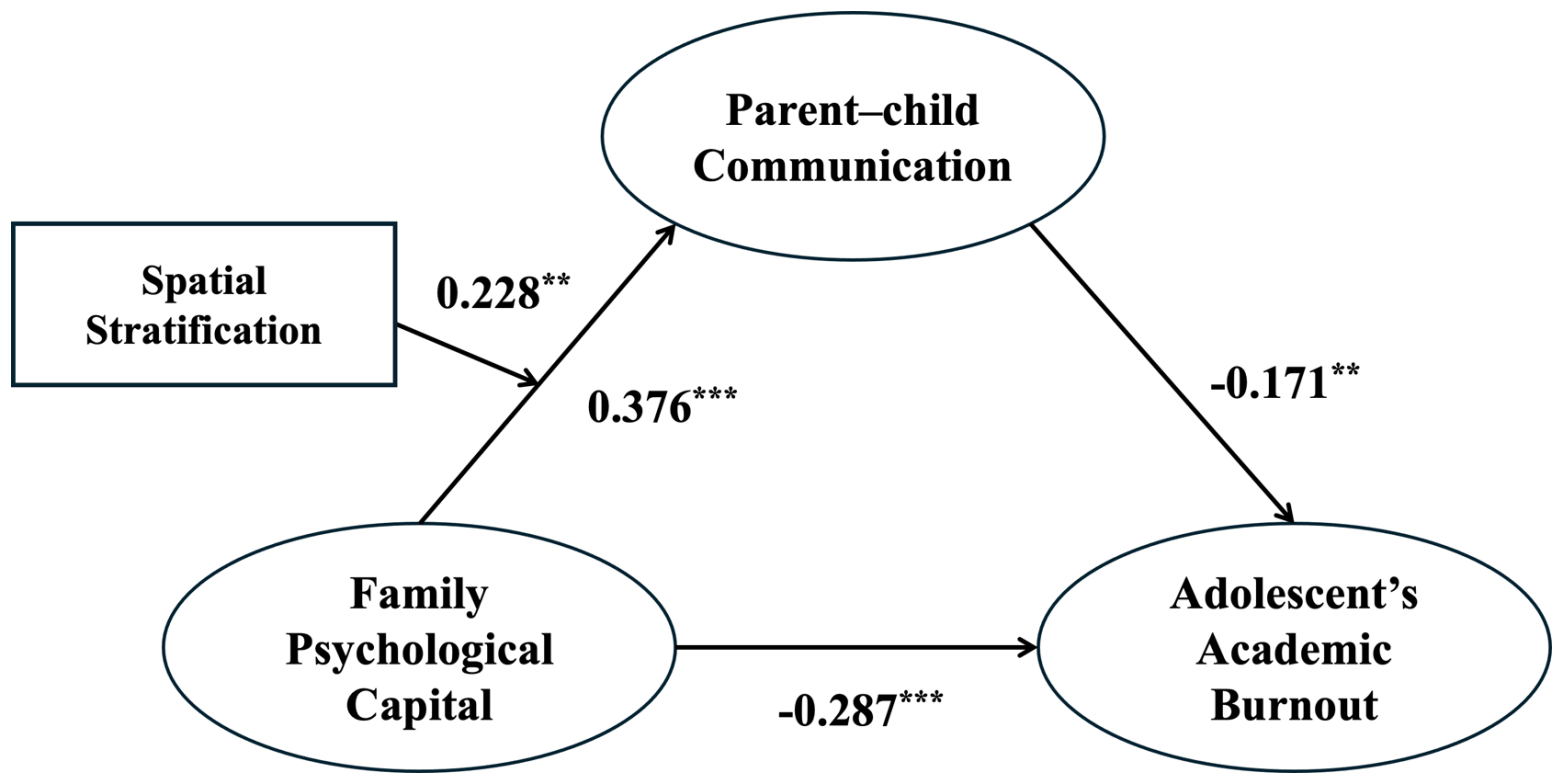
Moderated mediation model with spatial stratification as the moderator.

The moderated mediation analysis (Table 8; Figure 5) revealed significant findings regarding the contextual role of spatial stratification. Family psychological capital significantly and positively predicted parent–child communication (*B* = 0.376, *p <* 0.001). Moreover, the interaction term between spatial stratification and family psychological capital, in significantly predicting parent–child communication (*B* = 0.228, *p <* 0.01), indicated the presence of a moderating effect.

**Table 8:** Moderated mediation model (spatial stratification).

Predictor variable	B	SE	t
Family psychological capital	0.376	0.063	5.95^***^
Spatial stratification	−0.687	0.072	−9.50^***^
Family psychological capital * spatial stratification	0.228	0.076	2.99^**^
Fitting indicators	*R*	0.77	
	*R^2^*	0.59	
	*F*	237.78^***^	

*p < 0.05, **p < 0.01, ***p < 0.001.

Simple slope analysis further elucidated that moderating pattern (Figure 5). When participants were stratified by spatial classification, family psychological capital had significant positive effects on parent–child communication in both suburban (*B* = 0.376, *p <* 0.001) and urban groups (*B* = 0.604, *p <* 0.001). Notably, the predictive strength was significantly enhanced in the urban cohort, thereby suggesting that the advantage conferred by family psychological capital is more pronounced in urban settings.

The index of moderated mediation for the pathway through parent–child communication was −0.039 (95% CI [−0.088, −0.007]), which excluded zero. That result confirms that spatial stratification significantly moderated the strength of family psychological capital’s indirect effect on adolescent academic burnout through parent–child communication, which confirmed H3b. In sum, spatial stratification significantly moderated the mediating pathway, with the indirect effect of family psychological capital being more pronounced in urban contexts than in suburban settings.

### 6.6 Unified moderated mediation model

To determine whether spatial stratification and community social capital each moderated the association between family psychological capital and parent–child communication when both moderators and their respective interaction terms were included in the same model, we estimated a unified moderated mediation model using PROCESS Model 9.

As shown in Table 9, the mediator model was statistically significant, *R*^2^ = 0.592, *F* = 143.386, *p* < 0.001. After accounting for community social capital and its interaction with family psychological capital, the interaction between family psychological capital and spatial stratification remained significant, *B* = 0.278, *p* = 0.004, 95% CI [0.091, 0.464]. In contrast, after accounting for spatial stratification and its interaction with family psychological capital, the interaction between family psychological capital and community social capital was not significant, *B* = 0.027, *p* = 0.332, 95% CI [−0.028, 0.083]. At the mean level of community social capital, the positive association between family psychological capital and parent–child communication was stronger among urban families, *B* = 0.656, *p* < 0.001, than among suburban families, *B* = 0.379, *p* < 0.001.

**Table 9:** Results of the unified moderated mediation model.

Outcome and predictor	B	SE	t	p	LLCI	ULCI
Panel A. Outcome: parent–child communication						
Family psychological capital	0.379	0.067	5.628	<0.001	0.247	0.511
Spatial stratification	−0.677	0.073	−9.307	<0.001	−0.820	−0.534
Community social capital	−0.043	0.031	−1.360	0.175	−0.104	0.019
Family psychological capital × spatial stratification	0.278	0.095	2.927	0.004	0.091	0.464
Family psychological capital × community social capital	0.027	0.028	0.970	0.332	−0.028	0.083
Model fit	*R^2^* = 0.592		*F* = 143.386	<0.001	-	-
Panel B. Outcome: adolescent academic burnout						
Family psychological capital	−0.287	0.058	−4.949	<0.001	−0.401	−0.173
Parent–child communication	−0.171	0.058	−2.958	0.003	−0.285	−0.058
Model fit	*R^2^* = 0.183		*F* = 55.801	<0.001	-	-

Moderator	Index	BootSE	BootLLCI	BootULCI
Panel C. Indices of partial moderated mediation				
Spatial stratification	−0.048	0.026	−0.107	−0.008
Community social capital	−0.005	0.005	−0.017	0.005

The index of partial moderated mediation was significant for spatial stratification, index = −0.048, BootSE = 0.026, 95% bootstrap CI [−0.107, −0.008], but not for community social capital, index = −0.005, BootSE = 0.005, 95% bootstrap CI [−0.017, 0.005]. The indirect association between family psychological capital and academic burnout through parent–child communication was stronger among urban families than among suburban families. No significant variation in this indirect association was found across levels of community social capital.

### 6.7 Sensitivity analysis of the family psychological capital composite

Paired-samples comparisons indicated that fathers reported slightly higher overall psychological capital than mothers, *M*_father_ = 133.30, *SD* = 14.63, *M*_mother_ = 129.97, *SD* = 14.18, *t* = 4.197, *p* < 0.001, *d*_z_ = 0.188. Fathers also reported higher self-efficacy, *t* = 2.769, *p* = 0.006, *d*_z_ = 0.124; resilience, *t* = 3.336, *p* = 0.001, *d*_z_ = 0.149; and hope, *t* = 4.755, *p* < 0.001, *d*_z_ = 0.212. No significant paternal–maternal difference was found for optimism, *t* = 0.172, *p* = 0.864, *d*_z_ = 0.008. All four significant differences remained significant after Holm correction, and their effect sizes were small.

To evaluate whether these paternal–maternal differences affected the substantive findings, all focal models were re-estimated using the alternative family psychological capital composite. In the mediation model, the indirect association through parent–child communication remained significant, indirect effect = −0.158, BootSE = 0.063, 95% bootstrap CI [−0.279, −0.032]. In the separate moderated mediation models, the interaction between family psychological capital and spatial stratification remained significant, *B* = 0.289, *p* = 0.003, and the index of moderated mediation remained significant, index = −0.050, 95% bootstrap CI [−0.114, −0.007]. By contrast, neither the interaction involving community social capital, *B* = 0.029, *p* = 0.337, nor its index of moderated mediation, index = −0.005, 95% bootstrap CI [−0.017, 0.006], was significant.

The unified model produced the same pattern. The interaction between family psychological capital and spatial stratification remained significant, *B* = 0.351, *p* = 0.004, and the corresponding index of partial moderated mediation was significant, index = −0.061, 95% bootstrap CI [−0.139, −0.011]. The interaction involving community social capital remained nonsignificant, *B* = 0.034, *p* = 0.347, as did its index of partial moderated mediation, index = −0.006, 95% bootstrap CI [−0.022, 0.006]. Thus, the substantive conclusions were unchanged when family psychological capital was constructed by separately standardizing and equally weighting paternal and maternal scores. Complete results are presented in Supplementary Tables S1, S2.

### 6.8 Summary

Our results systematically validate the complex mechanisms through which family psychological capital influences adolescents’ academic burnout. First, a significant negative relationship was established between family psychological capital and adolescents’ academic burnout, meaning that higher family psychological capital can significantly decrease adolescents’ academic burnout. Second, parent–child communication was identified as a partial mediator in that relationship. Third, spatial stratification demonstrated significant moderating effects, with the mediating pathway being significantly stronger in urban than in suburban areas. However, community social capital did not exhibit the hypothesized moderating effect on the relationship between family psychological capital and parent–child communication. Collectively, those findings substantiate a moderated mediation framework wherein family psychological resources operate through communication processes, contingent upon spatial context but not community social resources.

In sum, regarding our four hypotheses, H1, H2, and H3b were supported by the data, while H3a was not.

## 7 Discussion

The divergent patterns in our results encourage a deeper investigation into the underlying reasons why spatial stratification moderates the familial psychological process but community social capital does not. To understand that nuanced interaction, in this section we examine the findings through two complementary theoretical lenses: a sociological perspective informed by social capital theory and a psychological perspective grounded in ecological systems theory. In our dual analysis, we aim to explain how structural and contextual factors differently shape the pathway from family resources to outcomes among adolescents.

Interpreting the findings through Putnam’s framework of social capital offers a sociologically grounded understanding of how family resources shape adolescents’ experiences of academic burnout. Putnam (33), bonding social capital comprises trust-laden, reciprocal ties that structure daily interactions within families. Those ties function as a collective asset that enables information to circulate, expectations to be coordinated, and mutual support to be enacted. Our results suggest that families with greater psychological resources are better positioned to sustain effective communication patterns. Such communication occurs when conversations are more regular, mutual expectations are more transparent, and everyday interactions are more aligned with shared understandings of academic effort and coping. In that sense, parent–child communication operates not merely as an interpersonal skill but as a medium through which bonding social capital is produced, maintained, and mobilized (48).

Spatial stratification’s significant moderating effect highlights how structural context shapes the utility and leverage of familial capital. In urban settings, characterized by concentrated resources and intensified competition and complexity, the family’s internal stock of capital becomes crucial. It functions as a key filter and buffer against structural pressures, which explains why its translation into effective communication yields greater returns in those areas (39, 52). That finding underscores how geographical inequality conditions the social efficacy of family resources.

Conversely, the lack of a moderating effect from community social capital suggests a notable decoupling between public and private spheres of capital in the urban context. The generalized trust and networks measured at the community level may represent a form of public, or “bridging,” capital that does not readily permeate the intimate, “bonding” capital dynamics within the family (33). In fast-paced, mobile urban environments, the family appears to operate as a relatively autonomous social unit, its internal capital functioning effectively regardless of the broader community. Perhaps the social resources available in the neighborhood are more superficial or instrumentally oriented. If so, the family microsystem would function as a relatively self-contained context for the academic coping process examined here, consistent with Bronfenbrenner's (14) emphasis on proximal processes within the immediate environment.

From a psychological perspective grounded in Bronfenbrenner’s (14) ecological systems theory, our findings illuminate how the dynamic interplay between proximal processes and distal environmental systems shapes adolescents’ development. The confirmed mediation through parent–child communication strongly supports the theory’s emphasis on the microsystem as the primary engine of development. The parent–child dyad constitutes a core proximal process (53), wherein sustained, progressively more complex interactions drive growth. Our results demonstrate that familial psychological capital fuels the quality of those interactions. In particular, parents’ hope, efficacy, resilience, and optimism enhance the frequency and warmth of reciprocal communication, which directly mitigates adolescents’ academic burnout (52). That dynamic aligns perfectly with the postulate that enduring forms of interaction in the immediate environment propel adolescents’ development.

The differential impact of the two moderators offers a nuanced view on how the exosystem and macrosystem influence microsystemic processes. Spatial stratification, as an exosystem factor that influences children via the family, shows how structural inequalities and competitive pressures fundamentally alter how family resources are used. In urban settings, the exosystem exerts greater pressure—for example, through competition at school, and access to information—thereby making the microsystemic process of communication more critical and amplifying the value of the psychological capital that sustains it. Conversely, the non-significant community social capital’s moderating role in the mesosystem (i.e., linkages between microsystems) indicates that, in the urban context that we studied, the connection between the community and family microsystems may be weaker than theorized. The measured aspects of community life (i.e., general trust and networks) may not actively penetrate or strengthen the specific proximal process of parent–child communication regarding academic stress (40, 41). That outcome suggests that in highly mobile, metropolitan environments, the family microsystem may operate independently from the immediate residential community.

Our findings generate a compelling dialogue at the intersection of sociology and psychology. Social capital theory and ecological systems theory are not mutually exclusive but offer explanatory frameworks at different levels. The former emphasizes the type of resources and structural conditions (e.g., forms of capital and spatial inequality), whereas the latter clarifies the specific contexts and processes through which those resources operate (e.g., microsystemic interactions and exosystemic pressures).

Our findings bear weight precisely at the convergence of those two frameworks. Family psychological capital, as a form of internalized social capital, exerts its influence by following the principles of proximal interaction from psychology (i.e., via the micro-process of parent–child communication) while being profoundly moderated by the structural context highlighted in sociology (i.e., spatial stratification). The absence of community social capital’s moderating effect jointly reveals a potential boundary shared by both theories in contemporary urban settings. When community ties become sparse or instrumental, their influence on highly intimate emotional processes within the family may be diminished, whether viewed as a resource network or developmental context. That theoretical interplay also promotes a more nuanced understanding of the results themselves—namely, structural factors (e.g., space) demonstrate a stronger moderating force than relational factors (e.g., community). That finding suggests that China’s rapid urbanization has prioritized the distribution of institutional resources (e.g., educational resources) over traditional neighborhood social networks (8, 10).

Consequently, family coping strategies, tending to focus inward, cultivate psychological resources instead of relying outward on community support. That tendency aligns with the social trend of increasing the independence of the “nuclear family,” so to speak, in urban life. Situating that conclusion in Shanghai clarifies its policy and social implications. As a Chinese megacity, Shanghai is an educational hub due to pressures of academic competition and residential stratification. The discovered “urban effect” and the paradox of generally low-quality communication but higher returns on capital among urban families directly reflect the real dilemma faced by many families in Shanghai in the race for elite education. Intense competition squeezes out time for relaxed parent–child communication; however, precisely for that reason, parents’ own stress resilience, optimistic beliefs, and other forms of psychological capital become invaluable. Recent education policies in Shanghai emphasizing double reduction (*shuang jian*) and promoting student mental health gain support from out findings from a family systems perspective. Policies aimed at reducing the burden on students should thus be implemented concurrently with measures that support parental psychological well-being and improve the family’s emotional ecosystem. Furthermore, community social capital showed no moderating effect. This indicates only that the form we measured (general trust and networks) was not linked to the family process, not that community resources are irrelevant. Whether ties that offer emotional support and reach into family life could strengthen urban family resilience, as Shanghai’s 15-min community life circle envisions, is a question for future research, not a conclusion of this study.

## 8 Conclusion

### 8.1 Significance

Our findings offer significant insights across theoretical, methodological, and practical domains. For theory, they advance ecological systems theory by delineating the conditions under which different environmental layers influence core family processes. The finding that a macro-structural factor (i.e., spatial stratification) demonstrates significant moderating power but a mesosystemic relational factor (i.e., community social capital) does not refine current understandings of contextual permeability in adolescents’ development.

For methodology, the contrasting results for the two moderators highlight the importance of specificity in ecological research. In demonstrating that all contextual layers are not equally salient for all familial processes, they urge researchers to critically select and justify moderators based on precise theoretical mechanisms instead of broad availability.

For practice, the results inform targeted support strategies. The efficacy of family psychological capital suggests that interventions should aim to bolster those internal resources, particularly for parents. However, its amplified effect in urban settings underscores a critical concern about equity, in that the same resource yields unequal returns based on geographic location. That dynamic highlights the necessity of structural interventions that mitigate the competitive pressures and resource disparities tied to spatial stratification, in order to ensure that family strengths translate to adolescents’ well-being more equitably across contexts. Concurrently, the non-significant role of general community social capital, in urging a re-evaluation of community-based programs, suggests a need to foster more specific, education-focused support networks that can directly engage with family dynamics.

### 8.2 Limitations

Several methodological and conceptual boundaries of our study warrant consideration when interpreting our findings. First and foremost, the cross-sectional nature of the data constitutes the main methodological limitation of this study. Because all variables were measured at a single time point, the hypothesized pathway from family psychological capital through parent–child communication to adolescents’ academic burnout should be interpreted as a statistically supported association rather than a causal sequence. Reverse or reciprocal influences, whereby adolescents’ burnout erodes the quality of parent–child communication or depletes parents’ psychological resources, cannot be ruled out by the present design. Longitudinal or cross-lagged panel designs, as well as intervention studies, are therefore needed to establish the temporal ordering and causal status of the relationships documented here. Beyond this design constraint, the study’s exclusive focus on urban families in China with high educational attainment also delimits the theoretical generalizability of the findings due to potentially overlooking how those mechanisms might operate in rural contexts or families with different socioeconomic characteristics. A further boundary concerns family structure: by design, our sample included only two-parent households, so the findings do not generalize to single-parent or blended families. This matters because family structure is itself linked to adolescent outcomes—adolescents in non-intact families report lower academic achievement and poorer mental health than peers in two-parent families (54). Extending the model to such families, and testing family structure as a moderator rather than holding it constant, is a useful direction for future research. Additionally, community social capital was reported by a single designated parent per household, which may introduce informant bias; future studies could collect reports from both parents to examine within-family agreement. Moreover, although we examined community social capital as a potential moderator, other contextual factors, including school climate or peer influence networks, remain unexplored yet theoretically relevant. Those boundaries suggest two directions for future research: investigating how digital environments might alter those psychological transmission processes and examining whether those patterns hold across different cultural contexts where spatial segregation and community social capital may operate through distinct mechanisms. Such investigations would further refine understandings of the ecological conditions under which family psychological resources most effectively promote adolescents’ well-being (55). Finally, although the sensitivity analyses indicated that the substantive findings were robust to separately standardizing and equally weighting paternal and maternal psychological capital scores, the observed paternal–maternal mean differences cannot be interpreted as direct evidence of gender inequality. The present study did not examine the social mechanisms underlying those differences or test measurement invariance of the psychological capital scale across fathers and mothers. Future research should test parent-gender measurement invariance and employ dyadic or latent-variable models to distinguish substantive parental differences from potential reporting or measurement differences.

## Data Availability

The raw data supporting the conclusions of this article will be made available by the authors, without undue reservation.
