# Supplementary material for: Family psychological capital and adolescent academic burnout in Shanghai: the moderating role of spatial stratification
Source: Front Public Health. 2026 Aug 19;14:1814753. doi: 10.3389/fpubh.2026.1814753 (PMC13533718; doi:10.3389/fpubh.2026.1814753)
Supplement: Supplementary file 1 [file Data_Sheet_1.PDF]

Supplementary Material: Sensitivity Analyses of the Family Psychological Capital Composite

S1. Analytical Procedure

The primary family psychological capital measure was constructed by summing paternal and maternal total psychological capital scores and subsequently standardizing the resulting family-level score. To examine whether this aggregation procedure was sensitive to systematic differences between father- and mother-reported psychological capital, two sets of sensitivity analyses were conducted.

First, paired-samples t tests were used to compare fathers and mothers within the same families on overall psychological capital and its four dimensions: self-efficacy, resilience, hope, and optimism. Because five paired comparisons were conducted, Holm-adjusted p values were calculated to account for multiple testing. Paired-samples effect sizes were expressed as d_z, calculated by dividing the mean paired difference by the standard deviation of the paired differences.

Second, an alternative family psychological capital composite was constructed by separately standardizing paternal and maternal total psychological capital scores and then averaging the two standardized scores:

$$FPC_{\text{alternative}} = \frac{z(FPC_{\text{father}}) + z(FPC_{\text{mother}})}{2}$$

The alternative composite was not standardized again. PROCESS Model 4, the two separate Model 7 analyses, and the unified Model 9 analysis were re-estimated using this alternative indicator. Bootstrap confidence intervals for indirect effects and indices of moderated mediation were based on 5,000 resamples.

Table S1. Paired Comparisons of Paternal and Maternal Psychological Capital

Dimension	Father, M (SD)	Mother, M (SD)	Mean difference	t	Raw p	Holm-adjusted p	d_z
Self-efficacy	35.92 (5.42)	35.05 (5.19)	0.87	2.769	.006	.012	.124
Resilience	35.99 (5.43)	34.90 (5.38)	1.10	3.336	.001	.003	.149
Hope	31.32 (4.87)	30.00 (4.35)	1.32	4.755	<.001	<.001	.212

Optimism	30.07 (4.74)	30.02 (4.72)	0.05	0.172	.864	.864	.008
Overall psychological capital	133.30 (14.63)	129.97 (14.18)	3.33	4.197	<.001	<.001	.188

S2. Results of the Sensitivity Analyses

As shown in Table S1, fathers reported significantly higher overall psychological capital, self-efficacy, resilience, and hope than mothers. No significant paternal–maternal difference was found for optimism. All statistically significant differences remained significant after Holm correction. The significant differences were small in magnitude, with d_z values ranging from .124 to .212, whereas the difference in optimism was negligible, $d_z = .008$.

Table S2 compares the focal results obtained using the original and alternative family psychological capital composites. The indirect effect through parent–child communication remained statistically significant under both operationalizations. Spatial stratification significantly moderated the first-stage pathway in both the separate Model 7 analysis and the unified Model 9 analysis, and the corresponding indices of moderated mediation excluded zero. In contrast, the interaction involving community social capital and its corresponding indices of moderated mediation remained nonsignificant.

Therefore, the direction and statistical significance pattern of the focal effects remained unchanged when paternal and maternal psychological capital scores were standardized separately and equally weighted.

Table S2. Robustness Analyses Using the Alternative Family Psychological Capital Composite

Model and focal effect	Original composite	Alternative composite
Model 4: X→M	$B = .718$, $SE = .031$, $p < .001$, 95%CI [.657, .779]	$B = .911$, $SE = .040$, $p < .001$, 95%CI [.833, .989]
Model 4: direct effect	$B = -.287$, $SE = .058$, $p < .001$, 95%CI [−.401, −.173]	$B = -.362$, $SE = .074$, $p < .001$, 95%CI [−.507, −.218]
Model 4: indirect effect	Effect = −.123, BootSE = .049, 95%CI [−.216, −.026]	Effect = −.158, BootSE = .063, 95%CI [−.279, −.032]
Spatial Model 7: X×W	$B = .228$, $SE = .076$, $p = .003$, 95%CI [.078, .378]	$B = .289$, $SE = .097$, $p = .003$, 95%CI [.099, .479]

Spatial Model 7: index of moderated mediation	Index = −.039, BootSE = .021, 95%CI [−.088, −.007]	Index = −.050, BootSE = .027, 95%CI [−.114, −.007]
Community Model 7: X×W	*B* = .023, *SE* = .024, *p* = .329, 95%CI [−.024, .070]	*B* = .029, *SE* = .030, *p* = .337, 95%CI [−.031, .089]
Community Model 7: index of moderated mediation	Index = −.004, BootSE = .004, 95% CI [−.013, .005]	Index = −.005, BootSE = .006, 95%CI [−.017, .006]
Unified Model 9: X×spatial stratification	B = .278, *SE* = .095, *p* = .004, 95%CI [.091, .464]	B = .351, *SE* = .121, *p* = .004, 95%CI [.114, .588]
Unified Model 9: X× community social capital	B = .027, *SE* = .028, *p* = .332, 95%CI [−.028, .083]	B = .034, *SE* = .036, *p* = .347, 95%CI [−.037, .104]
Model 9: partial moderated mediation index for spatial stratification	Index = −.048, BootSE = .026, 95%CI [−.106, −.008]	Index = −.061, BootSE = .033, 95%CI [−.139, −.011]
Model 9: partial moderated mediation index for community social capital	Index = −.005, BootSE = .006, 95%CI [−.017, .005]	Index = −.006, BootSE = .007, 95%CI [−.022, .006]
